# Assessment of a conduction-repolarisation metric to predict Arrhythmogenesis in right ventricular disorders^☆^

**DOI:** 10.1016/j.ijcard.2018.05.063

**Published:** 2018-11-15

**Authors:** C.A. Martin, M. Orini, N.T. Srinivasan, J. Bhar-Amato, S. Honarbakhsh, A.W. Chow, M.D. Lowe, R. Ben-Simon, P.M. Elliott, P. Taggart, P.D. Lambiase

**Affiliations:** a Barts Heart Centre, Barts Health NHS Trust, West Smithfield, London EC1A 7BE, UK; b Institute of Cardiovascular Science, University College London, Gower Street, London WC1E 6BT, UK

**Keywords:** Ventricular Tachycardia, Repolarization, Ablation, Risk stratification

## Abstract

**Background:**

The re-entry vulnerability index (RVI) is a recently proposed activation-repolarization metric designed to quantify tissue susceptibility to re-entry. This study aimed to test feasibility of an RVI-based algorithm to predict the earliest endocardial activation site of ventricular tachycardia (VT) during electrophysiological studies and occurrence of haemodynamically significant ventricular arrhythmias in follow-up.

**Methods:**

Patients with Arrhythmogenic Right Ventricular Cardiomyopathy (ARVC) (*n* = 11), Brugada Syndrome (BrS) (*n* = 13) and focal RV outflow tract VT (*n* = 9) underwent programmed stimulation with unipolar electrograms recorded from a non-contact array in the RV.

**Results:**

Lowest values of RVI co-localised with VT earliest activation site in ARVC/BrS but not in focal VT. The distance between region of lowest RVI and site of VT earliest site (D_min_) was lower in ARVC/BrS than in focal VT (6.8 ± 6.7 mm vs 26.9 ± 13.3 mm, *p* = 0.005). ARVC/BrS patients with inducible VT had lower Global-RVI (RVI_G_) than those who were non-inducible (−54.9 ± 13.0 ms vs −35.9 ± 8.6 ms, *p* = 0.005) or those with focal VT (−30.6 ± 11.5 ms, *p* = 0.001). Patients were followed up for 112 ± 19 months. Those with clinical VT events had lower Global-RVI than both ARVC and BrS patients without VT (−54.5 ± 13.5 ms vs −36.2 ± 8.8 ms, *p* = 0.007) and focal VT patients (−30.6 ± 11.5 ms, *p* = 0.002).

**Conclusions:**

RVI reliably identifies the earliest RV endocardial activation site of VT in BrS and ARVC but not focal ventricular arrhythmias and predicts the incidence of haemodynamically significant arrhythmias. Therefore, RVI may be of value in predicting VT exit sites and hence targeting of re-entrant arrhythmias.

## Highlights

• A new Repolarisation Vulnerability Index (RVI) metric integrates dynamic changes in conduction and repolarisation.
• RVI predicts the earliest endocardial activation site of VT to a high degree of accuracy in Brugada Syndrome and ARVC.
• It also identified a population of patients more likely to develop haemodynamically unstable VT/VF over a 10 year follow-up period.
• RVI has the potential to guide ablation strategies in patients with unmappable VT.

## 1 Introduction

**Fig. 1:**
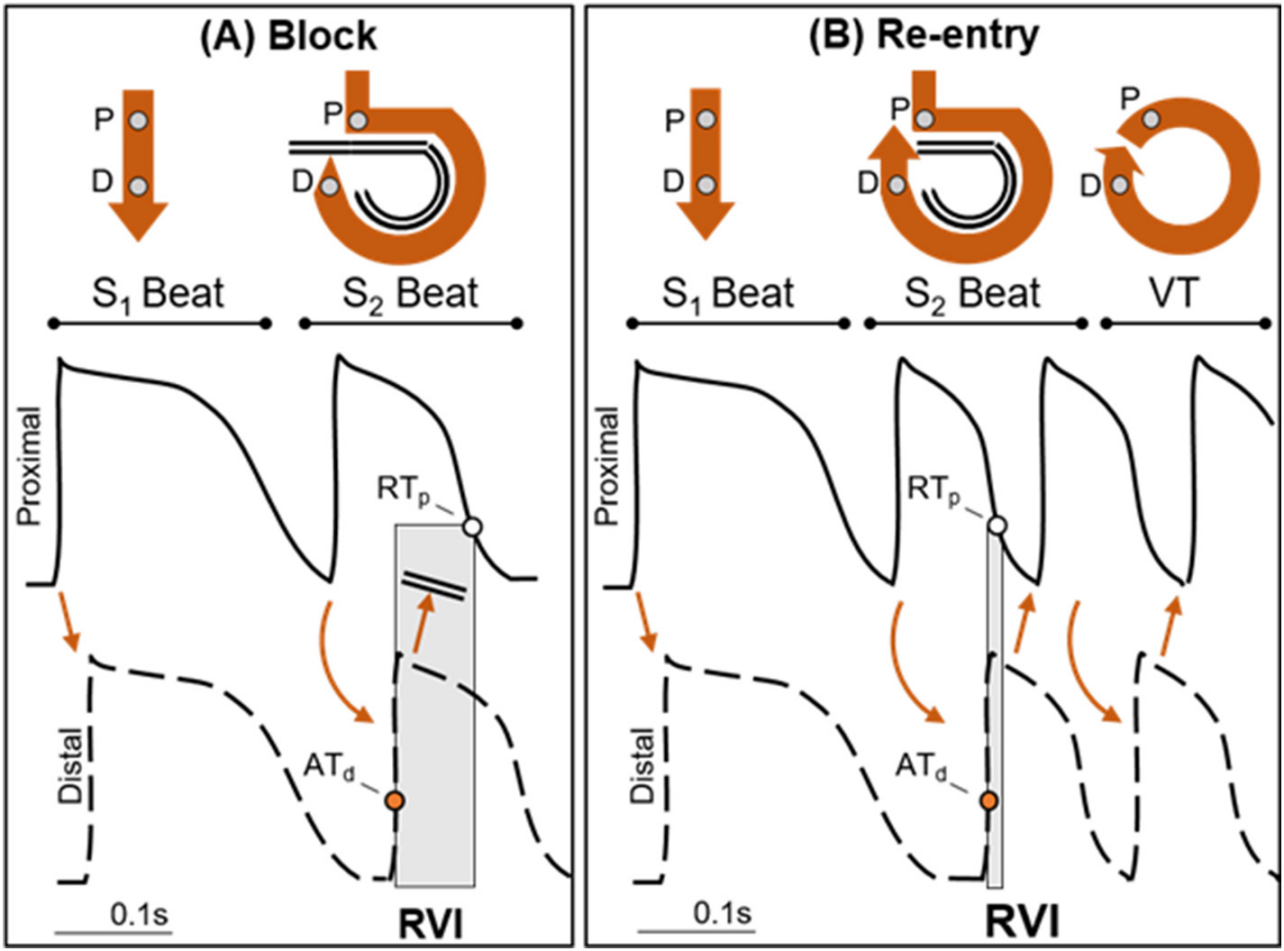
The RVI metric in the case of block (A) and re-entry (B). Stylised action potentials are shown during steady state pacing (S1) followed by a short coupled premature beat (S2). As shown in the top panels, the wave-front travels from a proximal (P) to a distal (D) site. During the S1 beat the wave-front conducts from the proximal to the distal site along a direct short path. However, during the S2 beat, the wave-front is blocked in the vicinity of the proximal site; it travels around a line of block and back towards the proximal site. In the first case (A), when the wave-front arrives at the distal site, the proximal site is still refractory, i.e. AT_d_ < RT_p_, and the re-entrant wave-front is blocked. In the second case (B), when the wave-front arrives at the distal site the proximal site has regained excitability, i.e. AT_d_ > RT_p_, enabling the wave-front to conduct and initiate re-entrant ventricular tachycardia (VT). The RVI is the interval between proximal repolarization (RT_p_) and distal (adjacent) activation (AT_d_) (shadowed area). Small or negative RVI values will enable a higher probability of re-entry.

A significant challenge remains in the prediction of ventricular tachycardia (VT) circuits, especially in disorders of diffuse fibrosis or where the functional characteristics of the substrate play a critical role in the initiation of re-entry. Recently, a novel spatial metric has been developed termed the *Re-entry Vulnerability Index* (RVI), used to locate regions of tissue susceptible to re-entry and VT initiation [1,2] (Fig. 1). This metric can theoretically be applied to predict the initiation site of monomorphic VT, as seen more commonly in Arrhythmogenic Right Ventricular Cardiomyopathy (ARVC), and polymorphic VT seen in Brugada Syndrome (BrS), as both initiate in the same manner around lines of block [[3], [4], [5]].

The RVI is based on previous experimental studies which quantitatively assessed the likelihood of excitation wavefront-waveback interactions between two points across a line of conduction block following a premature stimulus [6], therefore providing valuable information to potentially guide ablation when haemodynamically stable VT cannot be induced. The algorithm has been shown to accurately identify the region of a macro re-entrant circuit in 2 animal models and a single clinical case of ischaemic cardiomyopathy [1,2,6]. Pilot simulations of RVI maps in a rabbit ventricular model [7] suggested that the metric was able to reliably identify exit sites associated with re-entrant circuits for different scar morphologies.

The aim of this study was to test the feasibility and potential of RVI mapping in two right ventricular conditions where functional block and re-entry play an important role: BrS and ARVC [8,9]. We hypothesised that: (i) Although both ARVC and BrS are thought to be predominantly epicardial diseases, conduction-repolarisation abnormalities can be detected endocardially such that VT earliest activation (EA) sites in the RV endocardium co-localise with sites of lowest RVI during short-coupled beats [10]; (ii) RVI predicts the propensity to haemodynamically significant ventricular arrhythmias during follow-up. We elected to analyse data from a retrospective series of Brugada and ARVC patients with long term follow-up who had undergone clinical and mechanistic EP studies utilising non-contact mapping of the right ventricle to test these hypotheses.

## 2 Methods

### 2.1 Ethics statement

The research was approved by University College London Hospitals Ethics Committee A (08/H0714/97) according to the 1975 Declaration of Helsinki. Prior written informed consent to participate in this study was obtained from all participants.

### 2.2 Patient selection

38 patients aged 18–65 years were prospectively recruited to participate. Inclusion criteria: Diagnosis of ARVC by modified task force criteria (including familial or genetic criteria) or BrS based on type I ECG pattern (either spontaneous or provoked) with one of the following: documented VF, polymorphic VT, syncope or a family history of SCD. Patients with benign RV outflow tract (RVOT) ectopy or VT were the control cohort. All patients underwent detailed assessment with resting ECGs, signal averaged ECGs (SAECG) and echocardiography/MRI, and had stopped anti-arrhythmic medication for 5 half lives pre-EP study.

Following electrophysiological study (EPS), all patients had at least 50 months of follow up (mean = 112 ± 19 months). Patients were seen annually with clinical assessment, resting ECG, 24 h tape or device interrogation and serial imaging if appropriate. A clinical arrhythmic event was defined as VF or haemodynamically unstable VT either terminating spontaneously or by anti-tachycardia pacing (ATP) /ICD shock.

The RVOT VT group had normal resting ECGs and SAECGs, only unifocal ectopy or VT, structurally normal hearts on echocardiogram/MRI and no family history of SCD. In order to minimize the likelihood of a patient with their first presentation of hitherto clinically silent cardiomyopathy or channelopathy being mis-assigned a diagnosis of benign RVOT ectopy, focal VT patients were excluded if they had a recurrence of ventricular ectopy/tachycardia post ablation. All RVOT patients included in this analysis had normal ECG and cardiac imaging with no recurrence post focal ablation and no progressive ECG or imaging changes over time.

### 2.3 Genetic testing

Whole blood samples were obtained from participating BrS and ARVC subjects. Genomic DNA was extracted using a commercially available DNA extraction kit (QIAamp DNA Blood mini kit, Qiagen). ARVC cases were screened for mutations in desmoplakin, plakoglobin, plakophilin-2, desmoglein-2 and desmocollin-2, and BrS cases for mutations in SCN5A.

### 2.4 Electrophysiological mapping

The procedure for non-contact mapping has been previously described [11]. The 64 electrode non-contact array (St Jude Medical, USA) was placed in the low RVOT to map the RVOT and RV body via the left or right femoral vein under conscious sedation and was positioned within 4 cm of the endocardial surface to obtain accurate non-contact unipolar electrogram data.

Programmed stimulation was performed from the RV apex. Three minutes of steady state pacing at 600 ms coupling intervals was followed by an electrical stimulation protocol, consisting of an 8 beat train of pulses (S_1_) at 600 ms coupling interval followed by a single, premature stimulus (S_2_). The S_1_S_2_ coupling interval was reduced sequentially from 400 ms by 10 ms until ventricular refractoriness (VERP). A VT stimulation protocol followed, consisting of an 8 beat drive-train from the RV apex at 600 and 400 ms baseline cycle length followed by S_2_ until VERP or 200 ms coupling interval was reached. This was repeated for each baseline drive-train adding an S_3_ and S_4_ until VERP or 200 ms as per Wellens protocol.

### 2.5 Prediction of earliest site of endocardial activation during VT

The non-contact mapping data consisting of 256 unipolar electrograms were collected with a recording bandwidth of 0–300 Hz in all cases and measurements made with a filter band width of 0.1–25 Hz. Electrograms were exported and analysed using semi-automated custom software in Matlab (The Mathworks Inc., MA, USA) [12]. During pacing, the time from pacing artefact to the steepest negative deflection (dV/dt)_min_ was used as the local activation time (AT), while the time from pacing artefact to the steepest maximum deflection (dV/dt)_max_ was used to measure repolarization time independently of the polarity of the T-wave [13].

The requirement for the initiation of a re-entrant circuit is that a wave-front of activation travelling along a line of block finds tissue that has already regained excitability, enabling its reactivation (see Fig. 1). The likelihood that this condition is met increases when the wave-front is slow (long AT) and the wave-back is fast (short RT). The RVI algorithm provides a point-by-point quantification of this principle, by measuring the difference between RT and AT at pairs of adjacent points throughout the myocardium.

**Supplementary Fig. 1:**
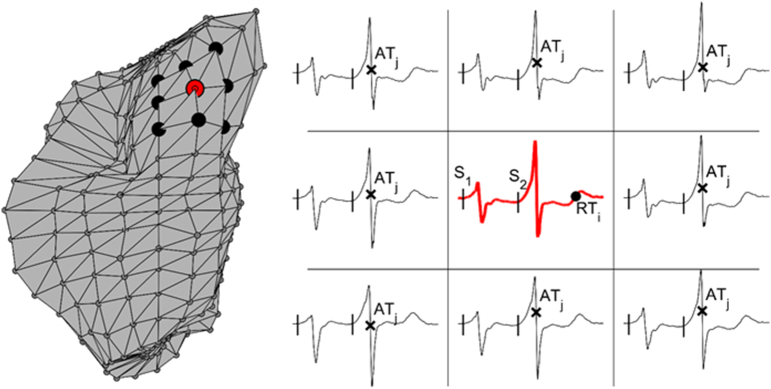
Calculation of RVI. Left: Neighbouring sites to an electrogram recording are identified within a pre-defined radius in a representative ventricular mesh from the clinical system. Proximal site i is shown in red, while distal neighbouring sites j are shown in black. Right: RVI at site i is the minimum difference between local RT at site i (black circle on red signal) and local AT at sites j (black crosses on black signals). RVI is computed in a premature beat following a short coupling S1S2 interval (vertical lines represent pacing artefacts).

The patient-specific location of the 256 ventricular sites from the clinical mapping system was used to create an RVI colour map. The RVI at each recording location *i*, RVI_i_, was calculated as the minimum difference between RT at site *i* (proximal) and AT at neighbouring sites *j* (distal) comprised within a 20 mm radius*,* AT_j_ [2] (Supplementary Fig. 1):

(1) $${\mathrm{RVI}}_{i}=\min_{j}\left({\mathrm{RT}}_{i}-{\mathrm{AT}}_{j}\right)$$

The lower this index, the higher the probability for a wave-front travelling along a line of block to find excitable tissue ahead of it and initiate a re-entry. RVI for prediction of earliest site of endocardial activation during VT was calculated during a beat following electrical stimulation at a short-coupled interval S_1_S_2,_ or where applicable S_2_S_3_, immediately preceding VT initiation. The site of earliest endocardial activation was identified from the analysis of all virtual unipolar electrograms and timed to the earliest minimum dV/dt on the isopotential map, ideally coinciding with a Q wave on the unipolar EGM. The distance (*D*_*min*_) between the region of lowest RVI (including the bottom 5% of all RVI values) and the region of earliest activation during VT (including sites that activated within 5 ms and that were located at <15 mm from the site of earliest activation) was computed for each patient.

### 2.6 Assessment of RVI as a marker of global vulnerability to re-entrant arrhythmia

Since vulnerability to re-entry is mechanistically related to low RVI, the lowest RVI value may not only identify the most vulnerable sites within a given heart, but it may also be used to assess the patient's susceptibility to re-entrant arrhythmias. In this respect, comparing RVI across patients may be problematic unless RVI mapping is performed at the same cycle length in all patients. In fact, since RVI is based on activation-repolarization dynamics it inevitably shows a strong cycle length dependency [12,14]. This implies that RVI may potentially reflect a difference in the cycle length of the premature beat rather than probability of arrhythmia initiation itself. To reduce RVI cycle length dependency, we proposed a patient-specific RVI correction which consists of subtracting the median RVI (RVI_med_) from the minimum RVI (RVI_10%_) [15]. This correction is based on the hypothesis that the median RVI mainly reflects the inter-subject heterogeneity and cycle length dependency of repolarization. This patient-specific metric, termed Global-RVI (RVI_G_), was defined as:

(2) $${\mathrm{RVI}}_{G}={\mathrm{RVI}}_{10\%}-{\mathrm{RVI}}_{\mathrm{med}}$$

Where RVI_10%_ denotes the 10th percentile of the RVI distribution, a robust estimate of the minimum RVI. Note that by definition Global-RVI, RVI_G_, is negative, with more negative values indicating higher risk of developing ventricular arrhythmia. This metric has been formally tested for independence from pacing cycle length between patients using contact epicardial sock recording during cardiac surgery [15]. Global-RVI was calculated from a single S_2_ beat in each patient with S_1_S_2_ coupling interval equal to 348 ± 17 ms.

### 2.7 Statistical analysis

The Wilcoxon rank-sum test was used to assess differences in D_*min*_. Group differences in global RVI were measured with the Kruskal-Wallis test for multiple comparisons followed by post-hoc Dunn-Sidak test for pairwise comparisons. *P* < 0.05 was accepted as statistically significant.

Standard box-plots were used to describe data distribution, where the central line is the median, the edges of the box are the first (Q1) and third (Q3) quartiles and the whiskers extend to the most extreme data points not considered outliers. Values lower than Q1–1.5*(Q3-Q1) and higher than Q3 + 1.5*(Q3-Q1) are considered outliers.

## 3 Results

### 3.1 Patient characteristics

The clinical characteristics and genetic results of all study subjects (13 BrS (54% male, age 53 ± 13 years), 11 ARVC (64% male, age 62 ± 12 years) and 9 focal RVOT VT (33% male, age 51 ± 8 years)) are shown in Table S1. In the BrS patients, 2 had a VF arrest at presentation and 1 had unexplained syncope; 3 were diagnosed with a BrS type 1 ECG incidentally and the remainder were diagnosed through family screening. Six (46%) had a positive VT simulation study with induced sustained VT/VF. None of the patients who were non-inducible at EPS had a clinical event. 5 BrS patients (38%) had an ICD; 2 were implanted for secondary prevention in the patients with VF arrests and 3 were implanted for primary prevention following inducible VT at EPS and other high risk features (1 further patient with inducible VT declined ICD).

In the ARVC patients, 1 had episodes of sustained VT at presentation, 7 were diagnosed after investigation for palpitations and 3 were diagnosed through family screening. As well as the patient with clinical VT at presentation, another 5 patients had haemodynamically significant clinical events at follow-up: episodes of symptomatic VT during follow-up that was either sustained in patients without a device (*n* = 2), or treated by ICD by ATP (*n* = 3). Four patients (36%) had a positive VT stimulation study; all 4, plus a further 2 who had been negative at EPS, were the patients with clinical events. Five (45%) ARVC patients had an ICD; this included the patient with VT on presentation who was implanted as secondary prevention. Four others had primary prevention devices.

During follow-up, patients with BrS did not routinely receive anti-arrhythmics, apart from one of the patients who had a VF arrest, who received a beta-blocker. Out of the 4 ARVC patients with VT, one received amiodarone and bisoprolol, two received bisoprolol and one received sotolol. Out of the 7 ARVC patients without VT, one received bisoprolol and the others did not receive anti-arrhythmics.

### 3.2 Prediction of VT locations

In 6 BrS (46%) and in 4 ARVC (36%) patients, VT was induced. A consistent feature seen in all cases was progressive slowing of conduction, followed by formation of an arc of functional conduction block on the RVOT endocardium. Lines of functional block evolved on a beat-to-beat basis. In 5 cases, the VT then degenerated to VF. Supplementary Fig. 2A, Supplementary Fig. 2B shows an example of a BrS patient with initiation of VT after development of an arc of functional block during a VT stimulation study (subject 6).

**Fig. 2:**
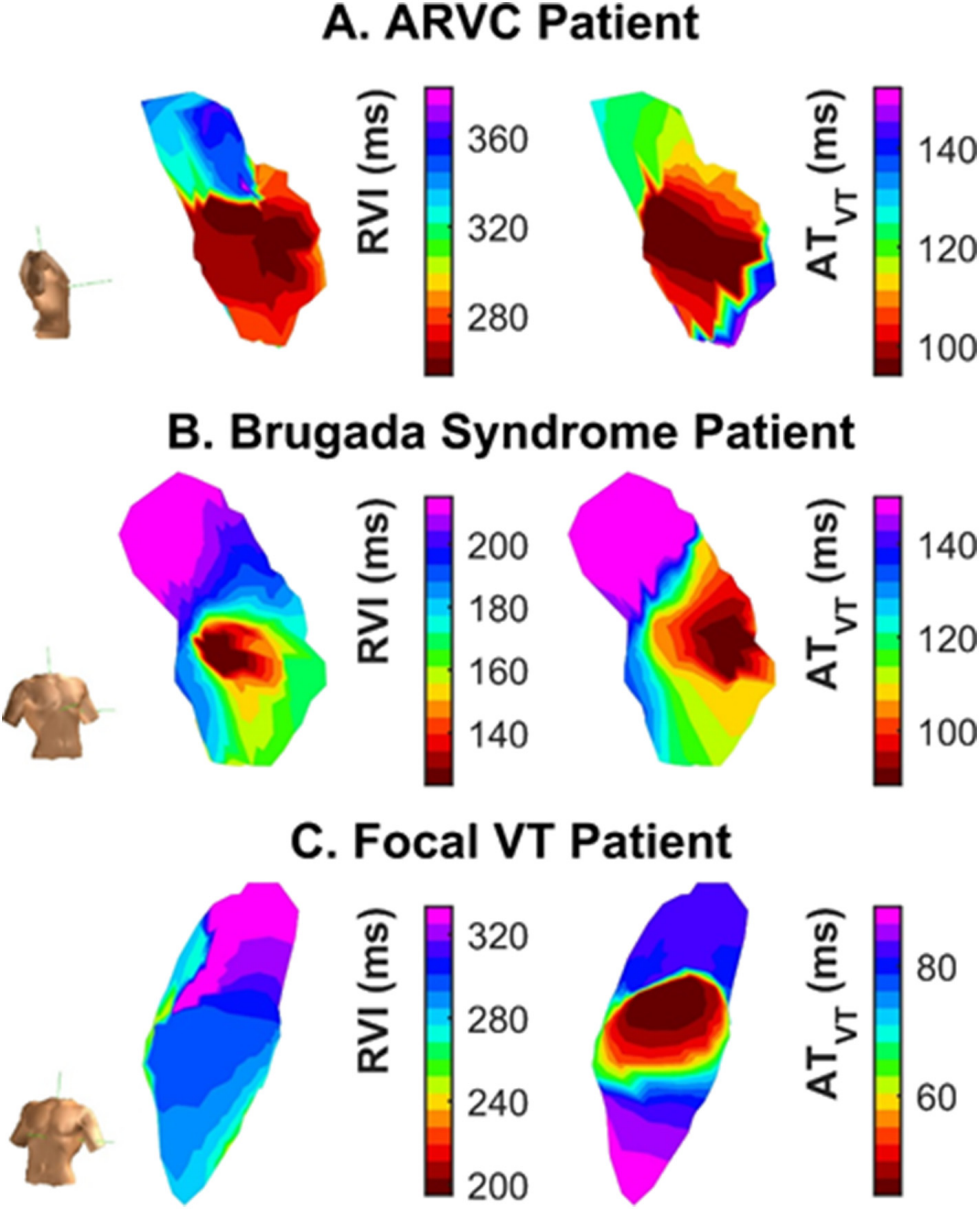
Spatial distributions of RVI measured before VT (left) and AT maps during VT (right) from representative patients with ARVC (A), BrS (B) and focal VT (C), demonstrating co-localisation of regions of lowest RVI (red) with earliest endocardial activation during VT (red) apart from in C.

Fig. 2 shows representative spatial distributions of AT during VT and RVI calculated during the S_1_S_2_ pacing protocol before VT. The shortest values of RVI, which represent sites of highest susceptibility to re-entry, co-localised with earliest activation point during VT in the ARVC (panel A) and BrS (panel B) patients where VT was initiated, but not in the patients with focal RVOT VT (panel C).

**Fig. 3:**
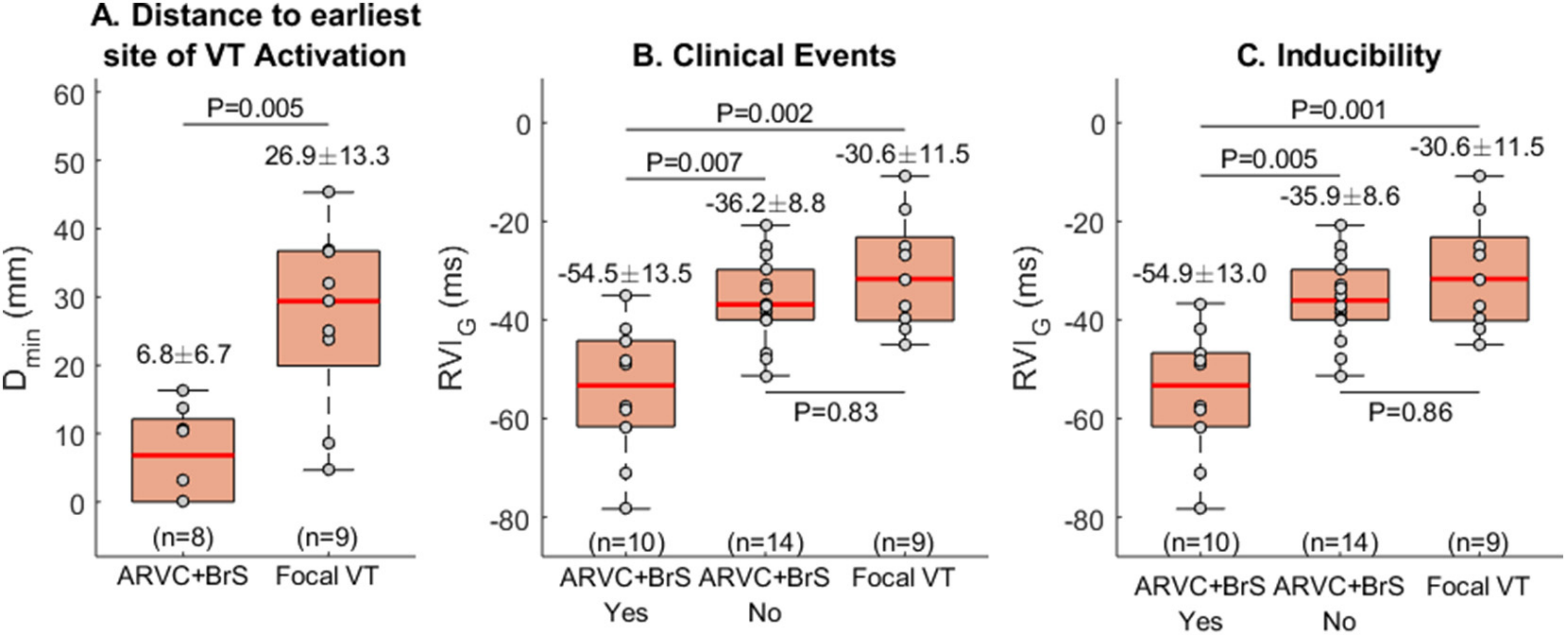
(A) The distance between the region of lowest RVI and the region of earliest activation during VT, *D*_*min*_, was significantly lower in BrS and ARVC than in focal VT. (B) BrS/ARVC patients with clinical VT events had lower Global-RVI, RVI_G_, than those without VT or than patients with focal VT. (C) Patients with ARVC/BrS with inducible VT had significantly lower RVI_G_ than those with no VT or than patients with focal VT. Mean ± standard deviation of data distribution are reported above each box, while the number of patients per group is reported on the bottom of the panel.

The distance between region of lowest RVI and region of earliest activation during VT, *D*_*min*_*,* was significantly lower in BrS and ARVC than in focal VT (6.8 ± 6.7 mm vs 26.9 ± 13. mm, *p* = 0.005) (Fig. 3A).

### 3.3 Low global RVI values predict likelihood of both clinical and inducible arrhythmias

The S_1_S_2_ coupling interval required for VT initiation was greater in those patients with clinical events (VF or haemodynamically unstable VT either terminating spontaneously or by anti-tachycardia pacing (ATP)/ICD shock) compared to those without clinical events (298.6 ± 54.1 ms vs 213.3 ± 4.7 ms, *p* < 0.05). There was no significant difference in the routine clinical VERP estimation (217.8 ± 22.5 ms vs 214.0 ± 14.5 ms, *p* = 0.61) in inducible versus non-inducible patients. Of the patients with ARVC/BrS, those with inducible VT had significantly lower Global-RVI, RVI_G_, than those with no inducible VT (−54.9 ± 13.0 ms vs −35.9 ± 8.6 ms, *p* = 0.005) or those with focal VT (−30.6 ± 11.5 ms, *p* = 0.001) (Fig. 3).

Patients were followed up for 112 ± 19 months. Clinical events in our cohort included VF (*n* = 2), haemodynamically unstable VT that terminated spontaneously (*n* = 4) or was successfully treated by ICD with anti-tachycardia pacing (ATP) (*n* = 3). Those with clinical VT events had lower Global-RVI, RVI_G_, than those without VT (−54.5 ± 13.5 ms vs −36.2 ± 8.8 ms, *p* = 0.007) or than those with focal VT (−30.6 ± 11.5 ms, *p* = 0.002) (Fig. 3). Receiver operating characteristic (ROC) analysis shows that Global-RVI was able to accurately discriminate between BrS/ARVC patients with and without clinical VT events (Sensitivity = 0.90, Specificity = 0.79, Accuracy = 0.83 for RVI_G_ < −40.8 ms) as well as between BrS/ARVC patients with clinical VT events and patients with focal VT (Sensitivity = 0.80, Specificity = 0.89, Accuracy = 0.84 for RVI_G_ < −43.9 ms) (Supplementary Fig. 3).

## 4 Discussion

The primary aim of this study was not to test the clinical utility of non-contact mapping in treating VT in BrS and ARVC but to use the data from patients with these conditions to test whether the RVI metric has potential value. The results of this study provide further evidence that RVI localizes regions of high susceptibility to conduction block and re-entry, with lowest RVI values identifying the earliest endocardial activation site of re-entrant VT but not the origin of focal arrhythmias. RVI is mechanistically related to re-entry vulnerability and can potentially be integrated into any electro-anatomical mapping system. This could be of value in predicting sites to target for ablation or assess risk. These principles could eventually be developed with non-invasive high density ECG recording advances in the future for risk stratification. Another potential application of the method is to differentiate between a focal and a reentrant mechanism based on the *D*_*min*_ and Global-RVI, which may have consequences for the choice of therapy.

Importantly, low Global-RVI is associated with a propensity for clinically significant ventricular arrhythmias in this series. Furthermore, patients for which VT was inducible showed a lower Global-RVI than patients for which VT was not inducible, therefore confirming the hypothesis that RVI is mechanistically related to re-entry susceptibility and offers the possibility of identifying the most vulnerable regions to ventricular arrhythmia in patients with hemodynamically unstable or non-inducible VT by simply performing electrical stimulation with a short coupling interval.

Although the pathophysiological processes in BrS and ARVC are different, they both exhibit a diffuse fibrotic substrate and may harbour a number of different conduction channels that support re-entrant circuits with multiple potential exit sites [4,9,16,17]. Local re-entry in these regions of scar can either initiate stable VT as in ARVC or degenerate into VF - this may be determined by the restitution properties of the tissue and probabilities of heterogeneous conduction-repolarisation changes promoting wavebreak [4,18]. Despite predominantly epicardial pathophysiology, the RVI metric successfully identified the earliest endocardial activation sites. This is most probably because the RV wall is only 3–4 mm thick, such that endocardial electrophysiological properties may give an indication of epicardial pathology. Furthermore, in the presence of epicardial disease it is plausible that the endocardium may be critical in promoting VT/VF by serving as the destination point for initiating re-entry after a line of block arises across the wall parallel to the epicardium. Previous work from our group has shown that both BrS and ARVC are distinguished by marked endocardial incremental conduction delays even in the absence of detectable fibrosis on imaging, indicating that functional electrophysiological changes are critical to the initiation of VT, especially early in the disease process [3,18]. In a detailed ex-vivo analysis of a BrS patient's heart, the arrhythmia originated in the endocardium of the RVOT, confirming that electrophysiological abnormalities extend beyond the subepicardial region [4].

Furthermore, risk stratification for the inherited arrhythmic conditions remains challenging in ARVC and BrS with a number of proposed risk markers [[19], [20], [21]]. The RVI algorithm was able to distinguish between patients with ARVC and BrS who had haemodynamically compromising clinical arrhythmias over nearly a decade of follow-up, as well as separating re-entrant from focal VT. This index could thus potentially be applied to risk stratify ARVC/BrS patients to target ICD prophylaxis or indeed give an indication of early cardiomyopathic process in RVOT ectopic cases. Development of software real-time signal processing could enable its integration into existing clinical EP systems to predict sites suitable for ablation.

### 4.1 Limitations

Although our cohort is small, we have a long period of follow-up data and our observed cardiac event rate is comparable with other large registries. For example, from the FINGER registry [19] the annual cardiac event rate in BrS patients was 7.7% in patients with aborted SCD (10.7% in our cohort) and 0.5% in asymptomatic patients (0.8% in our cohort). In a registry of ARVC patients [22], 48% had events over a 3 year follow-up, i.e. 16% annual event rate, irrespective of their initial presentation (5.8% in our cohort).

Simultaneous multi-site cardiac mapping was performed using a non-contact mapping system that has been proven to be accurate for both activation and repolarization measurements within 4 cm of the array [11,23]. Non-contact mapping has been widely used in the mapping of VT, and has been validated for use in the RVOT [23]. However, future studies using novel multi-polar contact catheters are needed to confirm our results.

We utilised a cohort of BrS and ARVC patients as these patients were attending for clinical EP studies using the array catheter. In patients with non-sustained or haemodynamically compromising VT, the use of global mapping enables activation, repolarization and scar information to be collected over the entire ventricle in a single beat, and therefore this strategy was critical to localise the VT earliest activation site and to perform RVI mapping.

We were only able to collect data from endocardial maps. Both BrS and ARVC have been described as predominantly epicardial diseases; however, the wall of the RVOT is extremely thin and previous studies have shown scar burden on endocardial mapping can inform prognosis and that endocardial ablation is successful in arrhythmia suppression in both these conditions [[24], [25], [26]]. The RVI algorithm was able to locate the site of earliest endocardial activation in VT and predict the probability of inducible and clinical ventricular arrhythmias in our study. We hypothesize that the RV wall at the site of earliest endocardial activation is either involved in the initiation of the re-entry or is close to the exit site as the RV wall is very thin (3–4 mm) [27]. However this remains speculative since confirmation of the VT isthmus by entrainment was not possible as the VTs were haemodynamically unstable, and current mapping technologies are unable to track electrical activity throughout the 3D cardiac structure to reveal the degree for epicardial-endocardial involvement in the initiation of VT in RV disorders. However, in 6 available ECGs the first VT beat had an ECG morphology consistent with an RV origin and the unipolar EGM (with a Q wave) at the site of earliest endocardial activation preceding or being on time with the QRS onset (Supplementary Fig. 4). We did not have MRI data available in the majority of patients to correlate the location of scar with the electroanatomical findings, which would be of interest in further studies. The ability of RVI to predict future clinical arrhythmic events is an interesting observation that needs to be formally tested in prospective cohorts.

## 5 Conclusions

RVI mapping proved effective in predicting endocardial VT earliest activation sites in this series of patients with right ventricular pathology as opposed to idiopathic ventricular ectopy. This supports the concept that RVI mapping could be of value in predicting sites of VT.

The following are the supplementary data related to this article.

Table S1 Table showing demographics of study participants. RWMA = regional wall motion abnormalities, MR = mitral regurgitation, TR = tricuspid regurgitation, LV = left ventricle. Table S1

**Supplementary Fig. 2A:**
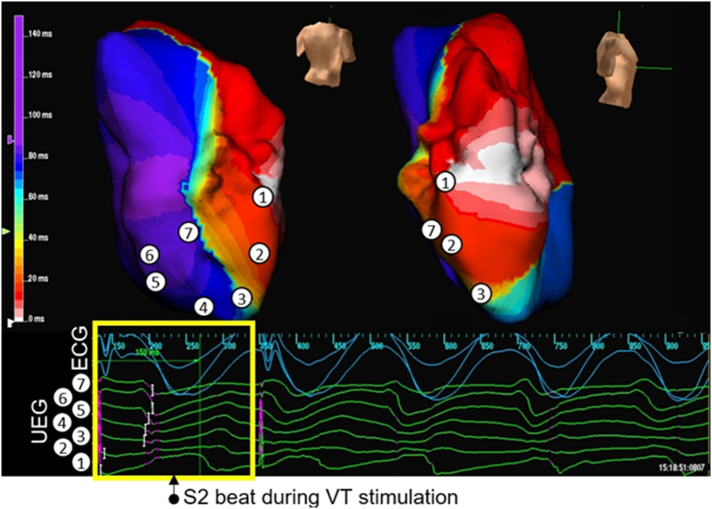
RV activation maps created with the Ensite array in a BrS patient (subject 6), showing initiation of VT. The RV is shown in posterior-anterior and right lateral views. The time scale of the isochrones is shown on the left of each map. Sampled areas are marked on the isochronal maps and corresponding virtual unipolar electrograms are shown below the map. An S2 beat during a VT stimulation study results in a line of functional block. This results in the initiation of a re-entrant circuit around this persistent line of functional block on the posterior RV endocardium.

**Supplementary Fig. 2B:**
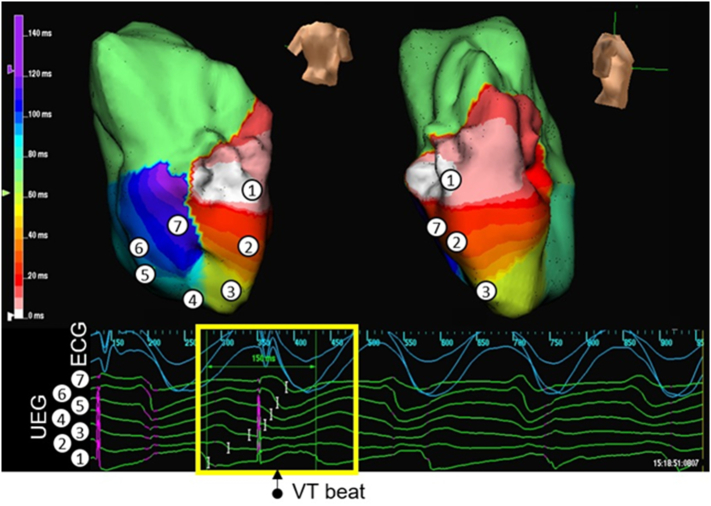
Pacing artefacts are shown in purple. The first pacing artefact shown is the S2 stimulus; the second would have been for an S3 beat but the stimulus failed to capture as VT had already commenced.

**Supplementary Fig. 3:**
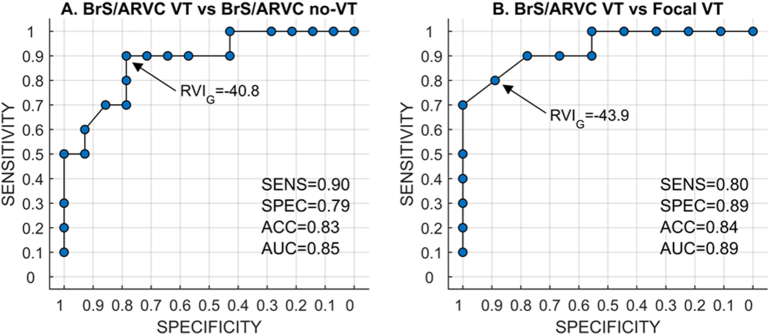
Receiver operating characteristic (ROC) analysis shows that Global-RVI was able to accurately discriminate (A) between BrS/ARVC patients with and without clinical VT events (Sensitivity = 0.90, Specificity = 0.79, Accuracy = 0.83 for RVI_G_ < −40.8 ms) as well as (B) between BrS/ARVC patients with clinical VT events and patients with focal VT (Sensitivity = 0.80, Specificity = 0.89, Accuracy = 0.84 for RVI_G_ < −43.9 ms).

**Supplementary Fig. 4:**
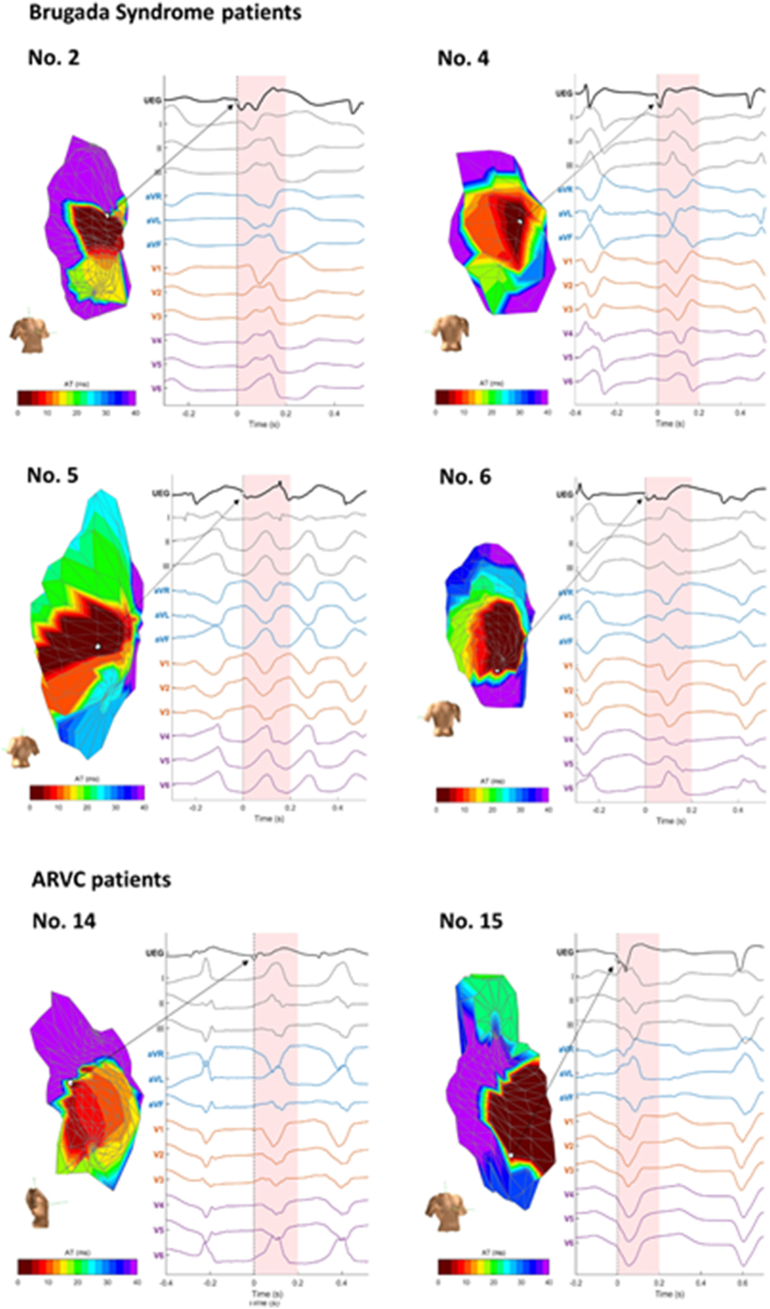
RV activation maps during VT initiation, with the point of earliest endocardial activation shown. The unipolar electrogram at this point is displayed, along with the 12 lead ECG. Cases where the 12 lead ECG was available are displayed, with 4 BrS patients and 2 ARVC patients. Case numbers correspond to those detailed in Table S1. The first VT beat has an ECG morphology consistent with an RV origin and the unipolar EGM (with a Q wave) at the site of earliest endocardial activation precedes or is on time with the QRS onset.

## Funding

The work was funded by a British Heart Foundation Project Grant PG/05/112, a University College London Hospitals Biomedicine National Institute for Health Research Grant and a Marie Curie European Training Fellowship (MO), Stephen Lyness Memorial Fund.

The authors report no relationships that could be construed as a conflict of interest.
