# Supplementary material for: Assessment of a conduction-repolarisation metric to predict Arrhythmogenesis in right ventricular disorders
Source: Int J Cardiol. 2018 Nov 15;271:75–80. doi: 10.1016/j.ijcard.2018.05.063 (PMC6152588; doi:10.1016/j.ijcard.2018.05.063)
Supplement: Table S1 — Table showing demographics of study participants. RWMA = regional wall motion abnormalities, MR = mitral regurgitation, TR = tricuspid regurgitation, LV = left ventricle. [file mmc1.docx]

| Subject No | Diagnosis | Inducible at EPS | Clinical VT/VF? | Follow-up time (months) | Age | Gender | Mutation | ICD | Macroscopic structural abnormalities on echo/MRI | SAECG | T-wave inversion V1-V3/epsilon waves | BrS type 1 V1-V3 persistently (P), intermittently (I) or with ajmaline (A)? | Positive family history? |
| --- | --- | --- | --- | --- | --- | --- | --- | --- | --- | --- | --- | --- | --- |
| 1 | BrS | Y | Y | 100 | 36 | M | N | Y | N | -ve | N | P | Y |
| 2 | BrS | Y | Y | 106 | 32 | M | N | Y | N | -ve | N | I | N |
| 3 | BrS | Y | Y | 137 | 48 | F | N | Y | N | -ve | N | I | Y |
| 4 | BrS | Y | N | 91 | 53 | M | N | N | N | -ve | N | I | N |
| 5 | BrS | Y | N | 81 | 64 | M | N | Y | RV - mildly dilated, hypokinetic | -ve | N | P | N |
| 6 | BrS | Y | N | 145 | 61 | M | N | Y | N | -ve | N | I | Y |
| 7 | BrS | N | N | 111 | 53 | F | N | N | N | -ve | N | A | Y |
| 8 | BrS | N | N | 111 | 49 | F | N | N | N | -ve | N | A | Y |
| 9 | BrS | N | N | 121 | 46 | F | N | N | N | -ve | N | A | Y |
| 10 | BrS | N | N | 131 | 52 | M | N | N | N | -ve | N | I | Y |
| 11 | BrS | N | N | 108 | 87 | F | SCN5A | N | Mild MR, mod TR | -ve | N | A | Y |
| 12 | BrS | N | N | 91 | 56 | M | N | N | N | -ve | N | P | N |
| 13 | BrS | N | N | 58 | 58 | F | N | N | N | -ve | N | P | Y |
| 14 | ARVC | Y | Y | 124 | 54 | M | N | N | RV RWMA, LV impairment | -ve | Y | N | N |
| 15 | ARVC | Y | Y | 135 | 77 | M | N | Y | N | -ve | Y | N | N |
| 16 | ARVC | Y | Y | 133 | 62 | F | N | Y | Mild biventricular dilatation, RV RWMA | -ve | Y | N | Y |
| 17 | ARVC | Y | Y | 113 | 71 | M | N | Y | RV RWMA, LV impairment | +ve | Y | N | N |
| 18 | ARVC | N | Y | 129 | 71 | F | N | Y | RV RWMA | +ve | Y | N | Y |
| 19 | ARVC | N | Y | 102 | 65 | M | Plakophilin-2 | N | RV RWMA, mild dilatation | +ve | Y | N | Y |
| 20 | ARVC | N | N | 129 | 75 | M | N | Y | RV RWMA | +ve | Y | N | N |
| 21 | ARVC | N | N | 117 | 71 | F | Desmoplakin | N | Mild biventricular dilatation | -ve | Y | N | Y |
| 22 | ARVC | N | N | 110 | 40 | F | N | N | N | +ve | N | N | N |
| 23 | ARVC | N | N | 105 | 45 | M | N | N | N | +ve | N | N | N |
| 24 | ARVC | N | N | 121 | 47 | M | Desmoglein-2 | N | Mild RV dilatation | +ve | Y | N | Y |
| 25 | Focal | Y | Y | 119 | 47 | M | N | N | N | -ve | N | N | N |
| 26 | Focal | Y | Y | 131 | 62 | F | N | N | N | -ve | N | N | N |
| 27 | Focal | Y | Y | 114 | 57 | F | N | N | N | -ve | N | N | N |
| 28 | Focal | Y | Y | 121 | 44 | F | N | N | N | -ve | N | N | N |
| 29 | Focal | Y | Y | 76 | 51 | F | N | N | N | -ve | N | N | N |
| 30 | Focal | Y | Y | 118 | 40 | F | N | N | N | -ve | N | N | N |
| 31 | Focal | Y | Y | 126 | 48 | M | N | N | N | -ve | N | N | N |
| 32 | Focal | Y | Y | 89 | 66 | F | N | N | N | -ve | N | N | N |
| 33 | Focal | Y | Y | 100 | 47 | M | N | N | N | -ve | N | N | N |

## **Tables**

**Table S1.** Table showing demographics of study participants. RWMA = regional wall motion abnormalities, MR = mitral regurgitation, TR = tricuspid regurgitation, LV = left ventricle.
